# The Contributions of Vestibular Evoked Myogenic Potentials and Acoustic Vestibular Stimulation to Our Understanding of the Vestibular System

**DOI:** 10.3389/fneur.2018.00481

**Published:** 2018-06-29

**Authors:** Sally M. Rosengren, James G. Colebatch

**Affiliations:** ^1^Neurology Department, Royal Prince Alfred Hospital, Camperdown, NSW, Australia; ^2^Central Clinical School, The University of Sydney, Sydney, NSW, Australia; ^3^Prince of Wales Hospital Clinical School and Neuroscience Research Australia, University of New South Wales, Sydney, NSW, Australia

**Keywords:** VEMP, otolith, sound, vibration, physiology, vestibular

## Abstract

Vestibular-evoked myogenic potentials (VEMPs) are short-latency muscle reflexes typically recorded from the neck or eye muscles with surface electrodes. They are used clinically to assess otolith function, but are also interesting as they can provide information about the vestibular system and its activation by sound and vibration. Since the introduction of VEMPs more than 25 years ago, VEMPs have inspired animal and human research on the effects of acoustic vestibular stimulation on the vestibular organs, their projections and the postural muscles involved in vestibular reflexes. Using a combination of recording techniques, including single motor unit recordings, VEMP studies have enhanced our understanding of the excitability changes underlying the sound-evoked vestibulo-collic and vestibulo-ocular reflexes. Studies in patients with diseases of the vestibular system, such as superior canal dehiscence and Meniere's disease, have shown how acoustic vestibular stimulation is affected by physical changes in the vestibule, and how sound-evoked reflexes can detect these changes and their resolution in clinical contexts. This review outlines the advances in our understanding of the vestibular system that have occurred following the renewed interest in sound and vibration as a result of the VEMP.

After its first description in 1992 (1), the vestibular-evoked myogenic potential (VEMP), a sound-evoked muscle reflex recorded from the sternocleidomastoid muscle, quickly showed clinical promise as an easy, non-invasive measure of vestibular function. Based on the animal data available at the time [e.g., (2)], it was suggested that the VEMP might be a saccular-dependent reflex. This was promising as it suggested that the saccule might now be tested in clinical settings, but the saccular origin was not certain. Sensitivity of the vestibular organs to sound was a well-established concept in animals [e.g., (3)], but its expression in humans was mainly limited to rare cases of the “Tullio phenomenon” (pathological activation of the vestibular system by sound). This condition was associated with a wide variety of pathologies prior to the discovery of superior canal dehiscence (SCD) as the usual underlying pathology (4). These two developments—the proposal of the VEMP as a new test of vestibular function and the demonstration of SCD as the likely cause of most cases of Tullio phenomenon—led to renewed interest in the effects of acoustic stimulation on the vestibular system. There followed an increase in the use of sound as a method of evoking reflexes in humans. Thus, aside from any clinical use, VEMPs and acoustic vestibular stimulation are interesting because they have contributed to our knowledge of the vestibular system, both in the normal ear and in vestibular disease. It is noteworthy that use of the VEMP has led to a renewed focus on vestibular sound sensitivity in animals, as it is more typical for animal research to motivate human studies. This review will describe the developments that have occurred in the field of vestibular research as a direct or indirect result of the introduction of the VEMP and the associated increase in popularity of acoustic vestibular stimulation.

Colebatch and Halmagyi became interested in sound-evoked, vestibular-dependent muscle reflexes due to much earlier reports by Bickford and colleagues, who discovered a vestibular reflex while looking for auditory-dependent responses on the scalp (5–7). Bickford et al. (5) recorded a response over the inion to very loud clicks, which was present in patients with profound hearing loss but preserved vestibular function, and absent in patients with both hearing and vestibular loss. This “inion response” was present only during contraction of the neck muscles, suggesting a myogenic (electromyographic, EMG) origin from the posterior neck muscles (5–7). Due to the difficulty in resolving the auditory and vestibular contributions to the inion response, Colebatch and Halmagyi (1) used a different recording site over the sternocleidomastoid (SCM) neck muscles. Moving the active electrodes away from the midline allowed investigation of the laterality of the reflex, while the use of SCM, a large superficial muscle, provided greater confidence in the origin and polarity of the reflex. They recorded a series of sound-evoked potentials from surface electrodes placed over the ipsilateral SCM neck muscles in a patient with Meniere's disease. The responses were best seen in the unrectified EMG and were only present during activation of the SCM. The response consisted of an initial positive peak followed by successive negative and positive peaks, and the earliest biphasic wave (p13–n23) was present before, but not after, vestibular neurectomy (Figure 1). It was also present in patients with sensorineural hearing loss and preserved vestibular function, indicating a solely vestibular origin. Subsequent, systematic investigations confirmed the vestibular-dependence of the early response, which is now termed the “cervical VEMP” or cVEMP (8).

**Figure 1:**
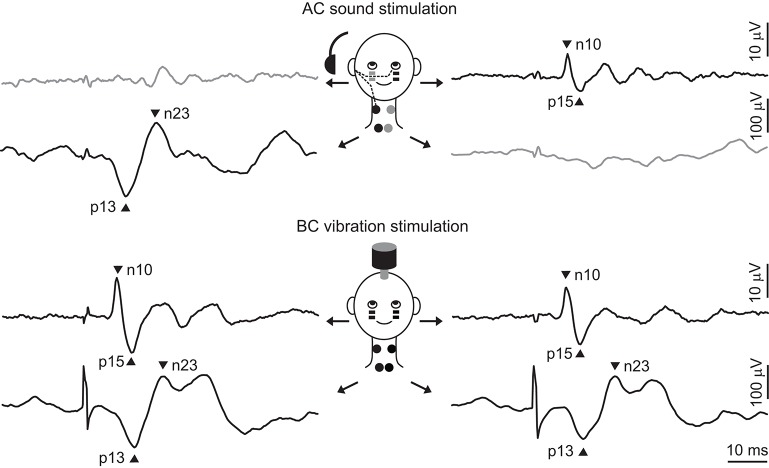
Typical cVEMPs and oVEMPs evoked by sound and vibration. The top part of the figure shows typical responses to AC sound stimulation of the right ear (recorded by black electrodes and shown by black traces). The projection to the SCM muscle is ipsilateral, producing a biphasic wave with peaks at 13 and 23 ms, while the projection to the IO muscle is contralateral, producing a response with peaks at 10 and 15 ms. There are usually no responses seen on the other side. The bottom part of the figure shows typical responses to BC vibration stimulation delivered to the forehead. Skull vibration activates both ears simultaneously and produces bilateral responses. In all traces there is a 20 ms pre-stimulus period and the stimulus is indicated by the brief artifact.

## VEMP-inspired animal studies

### Vestibular activation by sound and vibration

At the time of the cVEMP description, there was some evidence that it might originate in the saccule. Sensitivity of the vestibular organs to loud air-conducted (AC) sound and bone-conducted (BC) vibration had long been known. As early as 1916, Richard applied high-intensity AC sound to guinea pigs whose cochleas had been removed and recorded body movements (9). Well-known early experiments on acoustic vestibular stimulation include those by Tullio (10), who documented sound-evoked eye, head, and whole body movements after fenestration of the semicircular canals of pigeons and rabbits, and von Békésy (11), who recorded small head movements in normal humans in response to tones that were so loud that subjects often experienced a temporary threshold shift and had tinnitus for days afterwards. Direct evidence of vestibular nerve activation by sound followed. For example, Mikaelian (12) recorded intensity-dependent, sound-evoked potentials from the vestibular nerve of deaf mice after canal fenestration. As a result of these, and other, studies, Young et al. (3) systematically investigated the sensitivity of all five vestibular organs to sound and vibration. They stimulated squirrel monkeys with AC sound and found that the resting discharge of vestibular afferents became phase-locked to the stimulus. Saccular afferents had the lowest phase-locking threshold to sound, around 106–119 dB sound pressure level (SPL), while units in the other vestibular organs responded to higher intensity sounds. Afferents from all organs responded to vibration, and the most sensitive afferents responded to accelerations as low as 70–80 dB below 1 g. Several studies subsequently provided additional evidence of saccular activation by sound [e.g., (2)].

Following the first publications of VEMPs, there was a series of new studies on vestibular activation by sound, many as a direct result of interest in the new reflex. In several studies in cats, McCue and Guinan (13–15) recorded selectively from the inferior vestibular nerve and found that irregularly-firing afferents had lower thresholds to sound than regular afferents. The preferred frequency of stimulation was between 500 and 1,000 Hz. Limited intracellular labeling demonstrated that the fibers arose from the saccule, and not the posterior canal, whose fibers share the inferior nerve. These studies provided important confirmatory evidence for saccular sound sensitivity. Around the same time, Murofushi et al. (16–18) performed a series of experiments in guinea pigs using 0.1 ms AC clicks. They recorded responses from both click-sensitive and insensitive vestibular afferents and performed extensive labeling to confirm the origin of sound-sensitive fibers. They found that irregular afferents in the posterior branch of the superior vestibular nerve (which were traced to the utricle and saccule) and in the inferior vestibular nerve (traced to the saccule) were activated by sound. Most of these afferents were sensitive to tilt, while none responded to yaw rotation.

**Figure 2:**
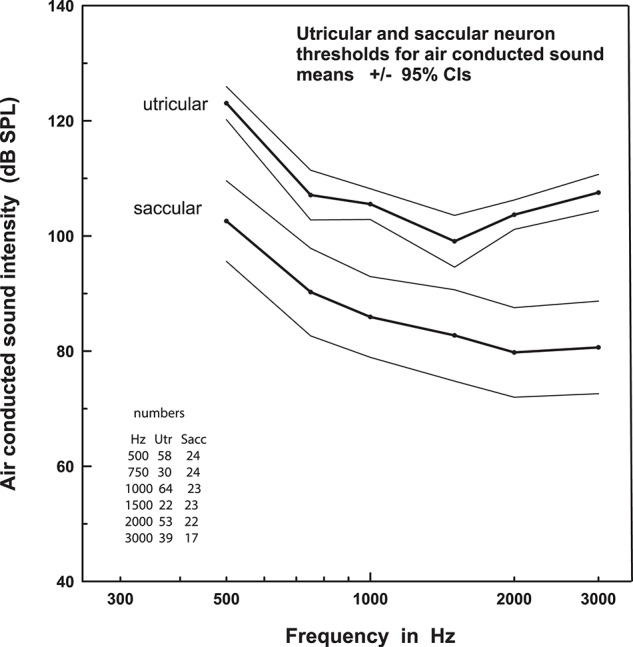
Thresholds to AC sound stimulation in the saccule versus utricle. This figure shows that the threshold for sound activation of saccular afferents is systematically lower than that of utricular afferents, across a broad range of frequencies. Data are mean ± 95% confidence intervals. Reprinted from Curthoys et al. (20).

Following on from these studies, Curthoys et al. (19) found that otolith afferents sensitive to AC sound could arise from either the saccule or the utricle. In a detailed tuning and sensitivity study, the authors showed that saccular and utricular afferents had broad tuning to AC sound between frequencies 500 and 3,000 Hz (20). Importantly, they reported that saccular afferents were on average 20 dB more sensitive to AC sound than utricular afferents (Figure 2), confirming the observations of Young et al. (3). Finally, Zhu et al. (21, 22) recorded AC click-evoked responses in the primary vestibular afferents of rats and found that clicks produced responses in twice as many otolith afferents (81%) as canal afferents (43%). Data on the probability of evoking a spike, the proportion of units showing strong responses, as well as response threshold, latency and duration all pointed toward a hierarchical effect, whereby otolith afferents from the inferior nerve (saccular afferents) had the strongest responses, followed by otolith afferents from the superior nerve (saccular and utricular afferents), followed by anterior, horizontal, and posterior canal afferents. There are clear differences in the results reported in these various studies, in part due to species and methodological differences, with disagreement on the degree of canal activation by AC sound. However, it appears that irregularly-firing otolith afferents, in particular those from the saccule, are most sensitive to AC sound, while semicircular afferents may also be activated, but to a lesser extent.

While initial studies used AC sound to evoke VEMPs, shortly thereafter it was reported that bone-conducted (BC) vibration applied to the skull was also an effective stimulus. Tendon hammers (23) and B-71 clinical bone-conductors normally used in audiometry (24, 25) were applied to the VEMP, and were particularly helpful in cases of conductive hearing loss. However, BC stimulation is more difficult to use, as additional amplification or external triggering is required, but the stimulus only increased in popularity after the description of the ocular VEMP (oVEMP), as BC stimulation turned out to be a more robust stimulus for this reflex (26). This increased clinical and experimental use of BC stimulation to evoke VEMPs again resulted in a renewed interest in the effects of the stimulus on vestibular afferents. Young et al. (3) had initially reported that the otoliths were *less* sensitive to vibration than canals. However, Curthoys et al. (20, 27) performed more extensive studies and found that otolith afferents were in fact *more* sensitive to vibration: 83% of irregular otolith afferents compared to 16% of irregular canal afferents responded to vibration. Similar to AC sound, it was predominantly the irregularly-firing otolith afferents that reacted to vibration (only 14% of regular otolith afferents were activated). Evidence suggested that afferents from both the saccule and utricle were sensitive to BC stimulation, with similar thresholds across the tested frequencies.

### Otolith projections to the neck muscles

**Figure 3:**
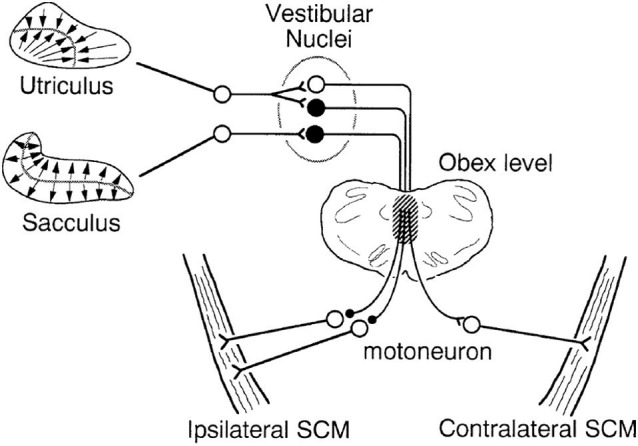
Schematic diagram of saccular and utricular projections to the SCM muscles in cats. Kushiro et al. (35) determined the projections to the SCM muscles from the otoliths for the first time. The saccule has an inhibitory projection to the ipsilateral SCM, while the utricle has a similar ipsilateral projection and an additional excitatory projection to the contralateral side. Filled neurons are inhibitory, and the open ones are excitatory. The hatched portion indicates the main pathway of the otolith-SCM connection. Reprinted from Kushiro et al. (35).

By the early 1990s, projections from the vestibular organs to the neck muscles had been extensively studied in cats, but there was no information on otolith projections to the SCM muscles in particular. It had been shown that the anterior canal had inhibitory projections to the flexors and excitatory projections to the neck extensors bilaterally, producing upwards head movements (28, 29), while the posterior canal had the opposite effect (28–30). The horizontal canal produced differential activity on the two sides of the neck, enabling the head to tilt sideways (31). The saccule inhibited the neck flexors and excited the neck extensors bilaterally, similar to the anterior canal (32), while the utricle had a differential effect on muscles on each side, similar to the horizontal canal (32, 33). These patterns of innervation produced compensatory head movements in the vertical (anterior and posterior canal and saccule) and horizontal axes (horizontal canal and utricle). To produce head rotation in the yaw direction, each vestibular organ also projects to the neck rotators, including the SCM muscles. At the time of the first VEMP reports, it was known that all three semicircular canals inhibited the ipsilateral SCM and excited the contralateral SCM (28, 34), but there was no information about the otolith projections. A study by Kushiro et al. (35) was initiated, in part, to fill this gap in the literature. They found that the utricle had similar disynaptic projections to the SCM as all three canals, but that the saccule had only an inhibitory projection to the ipsilateral muscle (Figure 3). The difference in projections between the vertical canals and saccule is likely to be due to the saccule contributing mainly to vertical head movements, while the vertical canals produce additional torsion of the head.

### Vestibular reflexes in animals

In 2005, Yang and Young described an animal model of cVEMPs in guinea pigs (36). They used clip-electrodes in restrained, alert guinea pigs and recorded sound-evoked myogenic reflexes bilaterally in the neck extensor muscles with stimulation of one ear. The response was biphasic and the first peak occurred at about 7 ms. The VEMP and caloric responses were abolished after topical administration of gentamicin, but the auditory brainstem response was preserved, confirming their vestibular-dependence. BC vibration can also produce cVEMPs in guinea pigs, though, similar to humans, the response evoked from each ear appears to be bilateral (37). Similar studies established an animal model of oVEMPs (38). Using subdermal needle electrodes, Yang et al. (38) recorded bilateral extraocular responses at an initial peak latency of ~3 ms in healthy guinea pigs using a BC vibration stimulus. Following application of gentamicin, a normal response was recorded on the side opposite the healthy ear only, demonstrating the same crossed projection as seen in humans.

## VEMP-inspired studies in normal volunteers

### Projections, laterality, and polarity of sound- and vibration-evoked vestibular responses

**Figure 4:**
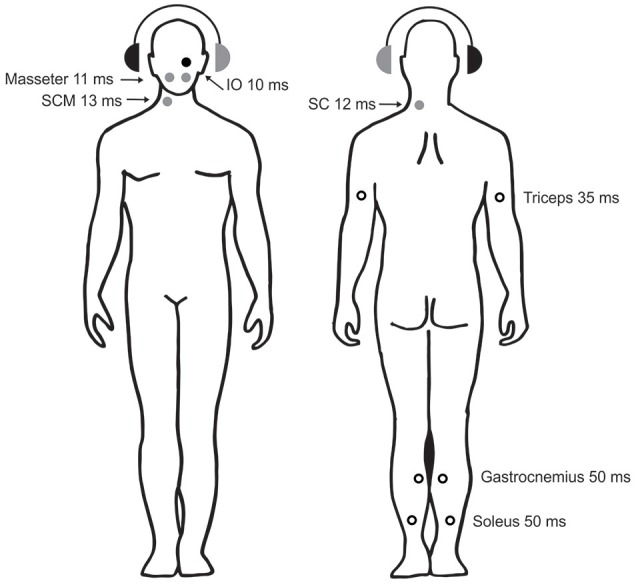
Sound-evoked reflexes in postural muscles. The circles show the sites, laterality, and approx. latencies of known AC-sound-evoked reflexes in postural muscles throughout the body. The right ear with the black headphone is the stimulated side. Solid circles show reflexes whose polarity has been confirmed with intramuscular recordings (black: excitatory reflexes, gray: inhibitory reflexes). Open circles show reflexes whose polarity has either not been definitively determined (triceps and gastrocnemius) or is known to depend upon head position (soleus). Data are from: IO—Weber et al. (39), masseter—Deriu et al. (40), SCM—Colebatch et al. (8), SC—Rosengren et al. and Camp et al. (41, 42), triceps—Cherchi et al. (43), gastrocnemius—Rudisill et al. (44), soleus—Bacsi et al. (45).

VEMP stimuli have properties that make them ideal for investigating otolith projections to postural muscles throughout the body and to the brain in humans (Figure 4). Sound and vibration are unique vestibular stimuli because they are extremely brief, can be delivered repetitively and cause little stimulus artifact. While traditional vestibular stimuli, such as rotation or translation and caloric irrigation, are very effective, they cannot readily be used to investigate short-latency effects. Sound and vibration have very short durations, of about 0.1 ms for an AC click and several milliseconds for an AC or BC tone burst, and allow detection of reflexes with onset latencies of a few milliseconds. Galvanic vestibular stimulation (GVS) can also be brief and applied repeatedly, but is typically associated with greater stimulus artifact. Sound and vibration are sufficiently mild to enable many repetitions and thus lend themselves to averaging, revealing effects that would otherwise be hidden. Air-conducted sound is also one of only few strictly unilateral vestibular stimuli. However, vestibular-dependent responses evoked using sound and vibration need to be distinguished from those mediated by the cochlea and (for vibration) the somatosensory system.

Many of the studies investigating vestibular reflex pathways described below used surface electrodes to record from the skin overlying the target muscle, a technique that is useful as it is non-invasive and often requires few stimuli, and can therefore be used in large numbers of volunteers. It is most effective when the muscle of interest is relatively superficial and located away from other muscles, as the signal can be assumed to originate primarily in that muscle. However, surface electrodes are prone to montage effects, being dependent upon the location of electrodes relative to the motor point and on the presence of other nearby sources. In contrast, intra-muscular recordings (e.g., single or multiple motor unit recordings) provide unequivocal information about the excitability changes underlying a reflex and are generally not affected by other nearby muscles (assuming the needle can be inserted accurately). They can also provide unique data about duration of any effect, however, they are invasive and require delivery of many more stimuli. A combination of these methods has led to elucidation of the projections described below (Figure 4).

#### Projections to the neck and face

In the neck muscles, the major sound-evoked response in SCM is an inhibition on the ipsilateral side, similar to the saccular projection shown in the cat. Surface responses first demonstrated the laterality of the reflex (Figure 1) (1, 8). The inhibitory nature of the response was demonstrated later by single motor unit studies showing a decrease or gap in probability of motor unit firing with a mean latency of 14.2 ms after stimulus onset (46, 47). Both surface and single motor unit responses to clicks showed that there were sometimes excitatory responses on the contralateral side [in up to 30% of cases (48)], which indicate that other vestibular afferents, with known excitatory projections to contralateral SCM, can also contribute (46). This is consistent with the animal research described above, suggesting that the utricle and, to a lesser extent, the semicircular canals can also be activated by AC sound.

Projections to the splenius capitis (SC) muscles have also been investigated (41, 42, 49–52). Splenius is an extensor and rotator located at the back of the neck and acts as an antagonist to the SCM on the same side. Surface responses in SC evoked by sound have been mixed and appear to depend on the method used to activate the muscle (extension versus rotation). This inconsistency may be due to the fact that SC is located amongst other muscles with differing actions and vestibular projections. In contrast, Camp et al. (41) and Rosengren et al. (42) recorded motor unit responses from within the SC muscle and found short-latency inhibitory responses at 12–14 ms on the contralateral side. Thus stimulation of one ear with AC sound produced inhibition in the ipsilateral SCM and contralateral SC, allowing the agonist muscle pair to rotate the head toward the stimulated ear. Projections to trapezius have also been examined, but responses were recorded with surface electrodes placed over the upper portion of trapezius in the posterior neck and so the precise origin of the bilateral inhibitory responses is not certain (53, 54). Studies of the original “inion response” described above suggest that there are also reflexes in the neck extensor muscles (5).

Vestibular-dependent reflexes in the masseter muscles, thought to be mediated by the vestibulo-trigeminal pathway, were first discovered using GVS and consisted of bilateral inhibitory reflexes at latencies of about 11 ms (55). Like the cVEMP, the masseter response increased in amplitude with increasing background EMG level, but was also modulated by tilt of the body. Similar responses were subsequently also produced by AC sound, and the response was thought to allow fine tuning of voluntary masseter movement (56, 40).

#### Projections to the eyes

The connectivity of the angular VOR has been well-established by animal studies (57). In contrast, projections from the otolith organs to the extraocular muscles are less clear. Otolith-ocular projections are more complex due to the variable polarization directions of otolith hair cells and the presence of the striola. In the available studies of whole nerve stimulation in cats, there is disagreement about the laterality and polarity of projections from the utricle to the vertical extraocular muscles (57, 58). Sacculo-ocular projections to the vertical recti and superior oblique muscles have been shown, but not specifically to the inferior oblique muscle, and these are generally weaker than all other vestibulo-ocular projections (57, 59). Projections to individual extraocular muscles in humans have been difficult to study for several reasons. Surface recordings are difficult because the muscles are close to each other and it can be hard to distinguish between electrical activity arising from the muscles or the retinal-corneal dipole. Vestibular-evoked eye movements are usually prohibitively large to perform intramuscular recordings safely. Sound and vibration stimuli have helped overcome some of these issues as they produce clear short-latency reflexes that can be distinguished from retinal-corneal dipole activity due to their amplitude and distribution. In addition, the accompanying eye movements are very small and allow intramuscular recordings.

VEMPs originating in the extraocular muscles were originally reported using BC-vibration and a referential electrode montage, and were largest in electrodes located beneath the eyes during up-gaze (60). Subsequent studies refined the recording methods and instead used a bipolar montage to record more selectively from the inferior extraocular muscles (61, 62). Using AC sound, oVEMPs were found to be clearly larger on the contralateral side (Figure 1) (61, 63). Bone-conducted stimulation produced bilateral responses in normal volunteers, but the response disappeared from the side ipsilateral to a vestibular lesion, demonstrating that the projection was contralateral (62). Although it is difficult to determine the reflex polarity and contributions of individual extraocular muscles using surface electrodes alone, early observations suggested that the response beneath the eyes might be an excitatory response of the inferior oblique (60). Weber et al. (39) demonstrated this *directly* by performing single motor unit recordings in the extraocular muscles of normal volunteers. They found an increase in motor unit firing evoked by BC vibration at 10.5 ms in the inferior oblique muscle, coinciding with the peak latency of the surface response at 10 ms. A similar response at 13.3 ms was found using AC stimulation and was seen only on the contralateral side. There was also an excitation in the inferior rectus muscles at 14.5 ms, indicating the presence of otolith projections to the vertical recti. It was not possible to record from the lateral recti. Attempts have also been made to selectively record from the lateral and superior muscles using surface electrodes (64, 65).

#### Projections to the trunk and limbs

Most of the knowledge we have of vestibular projections to the trunk and limbs comes from studies using GVS, as it is a strong stimulus that produces clear responses, and the reflexes are far enough from the stimulating electrodes to prevent artifact. Nashner and Wolfson (66) were the first to demonstrate that body sway produced by GVS was accompanied by reflexes in the full-wave rectified EMG of the leg muscles. They recorded changes in EMG in gastrocnemius and tibialis anterior at a latency of about 100 ms. With the cathode over the left mastoid and the head facing to the left subjects swayed forwards; the EMG in tibialis anterior increased and activity in gastrocnemius decreased. The opposite pattern was seen when the stimulus polarity was reversed and with opposite head rotation (66). The effect of head position was subsequently confirmed by others and is now considered characteristic of vestibular-dependent postural responses (67, 68). Studies using higher levels of current have shown that the vestibulo-spinal reflex in the tibialis anterior and soleus muscles is at least biphasic, with short- (SL) and medium-latency (ML) components of opposite polarity (69). The reflexes are also present only when the muscles are used for posture, and are absent during isometric contraction. Given the long initial latency of about 50 ms, compared to the fast conduction time along spinal pathways, there is likely to be significant central processing of the response before it reaches the postural muscles (69). Similar reciprocal EMG responses can also be recorded using GVS in other leg muscles, such as soleus (67, 68), muscles of the trunk (70), and in arm muscles (71).

**Figure 5:**
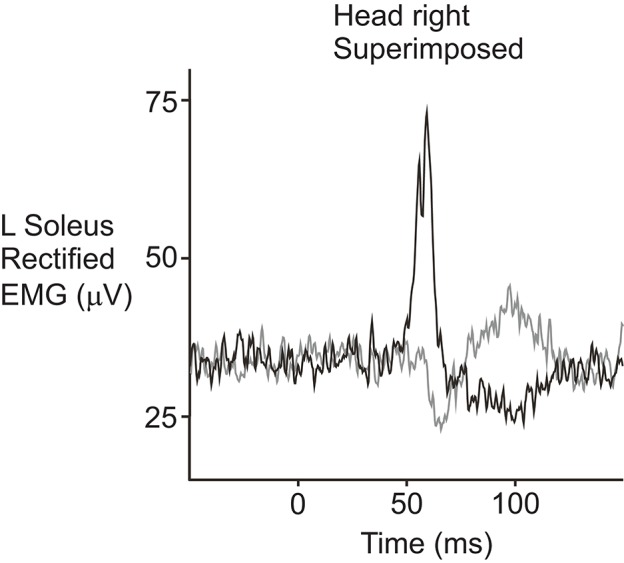
Sound-evoked reflexes recorded from the soleus muscle in a normal volunteer. The reflexes were recorded from the left soleus muscle while the subject stood with the head facing over the right shoulder. AC clicks delivered to the right ear (black trace) produced an initial increase in rectified EMG activity followed by a decrease in activity, similar to responses evoked by cathodal galvanic stimulation. Stimulation of the left ear (gray trace) produced responses with the opposite polarity. Reprinted from Watson and Colebatch (72).

Several studies have investigated the projections to the limbs using sound and vibration stimuli. Bickford et al. (5) first reported that acoustic stimuli produced responses in the arm and leg muscles. Later studies showed that AC sound stimulation produces responses in the soleus muscles of the leg that are similar in morphology, latency and polarity to those produced by cathodal stimulation (72). That is, stimulation of the right ear during head rotation to the right produced an initial excitatory response at ~50 ms in soleus (Figure 5). However, AC-evoked responses are much smaller than those evoked by GVS. When AC and GVS stimuli are matched in strength for their ability to evoke cVEMPs of equal size, the AC-evoked vestibulo-spinal reflexes are smaller and the ML response is often absent, consistent with saccular projections to the lower limbs being weak in normal subjects (45). Small responses to sound stimulation have also been recorded bilaterally in gastrocnemius at about 50 ms, but with the head facing forwards instead of to the side (44). Two studies have reported sound-evoked reflexes in the triceps muscles of the arms (43, 73). The response starts at about 35 ms with a positivity and can be seen on both sides when the arms are used to support body weight. The triceps responses are smaller than those in SCM and have a lower response rate.

#### Projections to the brain

A vestibular-dependent, sound-evoked response of possible brainstem origin was first described by Mason et al. (74). The authors recorded a surface negativity with a peak latency of ~3 ms (N3) during conventional auditory brainstem response (ABR) recordings in patients with severe hearing loss. The N3 potential was present only at very high intensities, above about 80–90 dB (74–76), and was correlated with the cVEMP (77). As the response was widely distributed and had a similar latency to the ABR, it was thought to originate from the vestibular brainstem nuclei (74, 76). The N3 potential was found in up to a quarter of deaf patients, but it has been difficult to record in normal subjects because it occurs at the same time as the ABR. Papathanasiou et al. (78–80) stimulated normal subjects with loud AC sound and recorded potentials from electrodes over the parietal cortex that were referred to the forehead. They found a negative potential at ~3–5 ms, but it was often obscured by the ABR. Murofushi et al. (81) delivered loud clicks and white noise to the same ear in attempt to reduce the impact of the ABR and found a response at 3.5 ms, which was correlated with the cVEMP, but not with hearing or caloric function.

Middle latency potentials have also been recorded from electrodes placed over the scalp using AC and BC stimulation (82, 83). Todd et al. (82) compared two stimuli: a 500 Hz stimulus (within the preferred frequency range of the vestibule) and a 5,000 Hz control stimulus (outside the preferred frequency range of the vestibule, and designed to activate only the auditory system). They found two groups of vestibular-dependent potentials: a positive/negative biphasic wave (P10/N17) maximal at the vertex (which was thought to be cortical in origin) and a negative/positive wave (N15/P21) maximal over the forehead (which was thought to be a vestibulo-ocular response). In contrast, the control stimulus produced only the well-established auditory responses (84). To confirm that the auditory system did not contribute to these responses, Rosengren et al. (85) recorded the same responses in a group of patients with severe to profound bilateral hearing loss. Although the P10/N17 potentials were originally thought to have a cortical origin, due to their wide distribution across the scalp and peak at the vertex, a subsequent study using source analysis showed that both sets of potentials actually originated near the eyes and were likely to be oVEMPs (86). There were thought to be residual, weaker contributions from neural sources such as the cerebellum, which was confirmed by a subsequent study (87). Long-latency potentials can also be evoked by vestibular stimulation with sound and vibration, and have been suggested to originate in the cingulate cortex (88, 89).

Sound-evoked cortical responses have also been examined with functional magnetic resonance imaging (fMRI). Clicks and short tone bursts activate areas of the frontal lobes (including the prefrontal cortex, frontal eye fields, and premotor cortex), the temporo-parietal lobes (including the superior and middle temporal gyri and the inferior parietal lobule), the cingulate cortex and the insula (90–92). These areas are similar to those activated by stimulation of the horizontal canal with caloric irrigation or the whole vestibular nerve using GVS. Activity is greater in the non-dominant hemisphere in both right- and left-handers (91, 92).

### Properties of sound- and vibration-evoked vestibulo-collic and vestibulo-ocular reflexes

VEMP studies have directly contributed to our knowledge of the vestibulo-collic (VCR) and vestibulo-ocular reflexes (VOR). Although it is sometimes said that sound and vibration are artificial vestibular stimuli, in fact the reflexes evoked by vibration are sensitive to the direction of stimulation (64, 65, 93–96), suggesting that, although the frequency of stimulation is outside what we normally consider the physiological range of vestibular afferents, the vibration-evoked responses are likely to share the same neural mechanisms as vestibular reflexes evoked by perturbations and are special examples of VCR and VOR.

#### Excitability changes underlying responses evoked by sound and vibration stimuli

##### Myogenic activity during cVEMPs

Single motor unit and mapping studies have increased our knowledge about the behavior of the muscles during the reflexes. Colebatch and Rothwell (46, 47) first recorded the activity of motor units in the SCM muscle in order to determine the nature of the cVEMP. They found short-latency changes in the probability of firing in 42 of 46 single motor units studied and the effect was always a decrease or gap in firing, indicating inhibition of the ipsilateral muscle. The latency of inhibition was about 14 ms, similar to the first peak of the surface response at ~13 ms, and the duration was very short (mean 3.6 ms, range 2–8 ms). There were also responses in the contralateral SCM in 9 of 16 single motor units, and these were excitatory in 8 of these units (latency ~12 ms, duration 2.3 ms). The fast onset and brief duration suggested that there was little temporal jitter in the pathways, although there was a large range of latencies in separate motor units (latency range 8–28 ms) (46). In contrast, the peak latency (p13) recorded with surface electrodes is less variable, as it represents the sum of all underlying motor units and averages out this variability. Consistent with this, the amplitude and duration of initial motor unit responses recorded from *single* motor units are not correlated with the size of the surface response, but *multiple* unit responses predict the surface response quite well (*r* = 0.66 and 0.69, respectively, for amplitude and duration) (93). Colebatch and Rothwell (46) also showed that more intense click stimuli were associated with longer periods of inhibition, circumstances which evoke larger amplitude surface responses.

**Figure 6:**
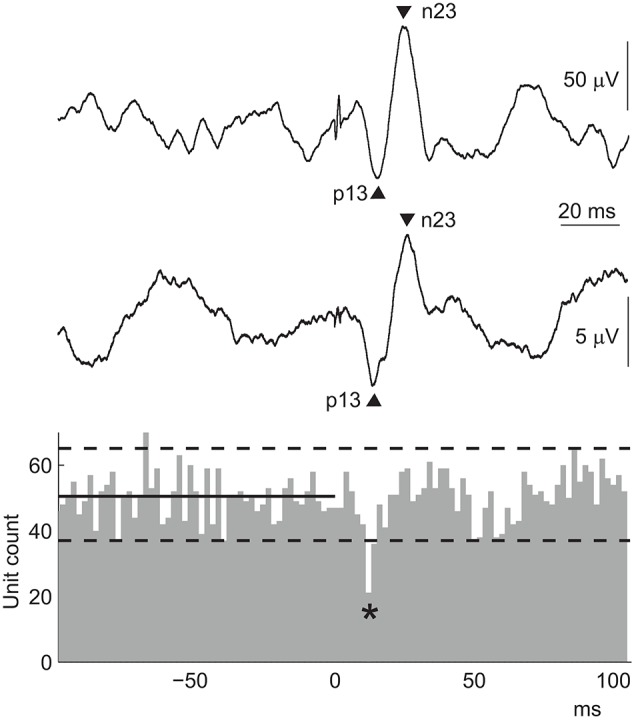
cVEMPs: surface versus motor unit responses. The peri-stimulus time histogram contains data from multiple motor units recorded simultaneously from the ipsilateral SCM in response to AC sound stimulation in a normal volunteer. The stimulus was delivered at 0 ms, and the solid and dashed lines show the median and 97.5 and 2.5 quantiles calculated over the 100 ms pre-stimulus period. The lower EMG trace shows the coinciding surface cVEMP from the same volunteer. The cVEMP is small because the muscle contraction during the intramuscular recording was weak to allow activation of only a few motor units. The top EMG trace shows a cVEMP from the ipsilateral SCM muscle recorded in the same subject under clinical conditions, i.e., with a moderate contraction and subsequently a larger amplitude. The cVEMP in both surface recordings is biphasic, with peaks at about 13 and 23 ms. The excitability change that produces this surface response is a single, brief inhibition of the muscle, shown by the decrease in unit count indicated by the asterisk in the histogram.

While there is a clear relationship between the p13 surface potential of the cVEMP and the initial motor unit response, the origin of the n23 surface potential is less clear. Rosengren et al. (93) found that an n23 potential could be seen in all surface recordings, but a corresponding increase in motor unit firing was present in only about half of the peri-stimulus time histograms (Figure 6). There was no relationship between the size of the n23 surface response and this inconsistent second motor unit response. Instead, both the p13 and n23 potentials were equally well correlated with the initial inhibition in motor unit activity (*r* = 0.55 and 0.60, respectively). This suggests that the inhibition is responsible for both peaks of the biphasic surface response, although recovery from the period of inhibition probably contributes to the n23 potential. In this way, the cVEMP surface response is like a “missing” or inverted compound muscle action potential (CMAP) (46). The CMAP is also a biphasic surface potential produced by a single change in motor activity, though in this case the reflex is excitatory. Interestingly, these responses are not always evident using surface averages of rectified EMG and, when present, paradoxically appear as increases in rectified activity (97).

In order to compare the properties of the cVEMP at different sites along and around the SCM muscle, Colebatch (98) first determined the location of the motor point: about 65% of the distance between the sternoclavicular joint and the mastoid process, i.e., just above the middle of the muscle belly. The reflex was largest when recorded over the motor point and had the shortest p13 latency, while the n23 latency was not affected by electrode position. This effect was confirmed in a subsequent mapping study (99), in which the cVEMP became systematically smaller and later as the electrode was moved away from the muscle belly toward the upper and lower SCM tendons. Single motor unit responses recorded at the upper, middle, and lower parts of SCM showed a similar latency effect, whereby the inhibition tended to occur earlier at the middle electrode site (median 12 ms) compared with the upper and lower sites (15 ms). The data from these studies shows that the sound-evoked VCR originates from the motor point and spreads gradually along the muscle. The p13 surface response behaves as a traveling wave, produced by the muscle inhibition as it begins at the motor point and moves along the muscle in both directions. The n23 potential behaves more like a combination of phenomena, potentially including a trailing dipole created following the propagating inhibition, a standing wave, and a small rebound in firing following the inhibition (99). The surface potential recorded at any point along the muscle will be a combination of these waveforms.

The inhibitory nature of the ipsilateral cVEMP explains the behavior of the surface response during changes in the background SCM muscle contraction. The cVEMP can only be recorded when the muscle is active because a gap in motor unit firing can only be detected if there is ongoing activity to pause. cVEMP amplitude also increases relatively linearly in amplitude as the tonic muscle activity increases (8, 100, 101). This is because a gap in firing will represent a greater change when the tonic activity is strong. The property of scaling with background activation (called “automatic gain compensation”) is expected when reflexes project to most units of the motor pool and is thought to apply to small reflexes of either polarity during weak to moderate strength contractions (102). Interestingly, the scaling effect interacts with the intensity of the stimulus (100, 101). Subjects with low cVEMP thresholds, in whom standard VEMP stimuli are relatively strong, are especially sensitive to the effects of muscle contraction. This is probably because each intensity stimulates a fixed proportion of tonically active motor units, and at higher intensities the number of motor units inhibited at each incremental EMG level will be proportionately greater than at lower intensities (101).

##### Myogenic activity during oVEMPs

Single motor unit recordings have also increased our knowledge about the vestibulo-ocular reflex. Only very few studies have investigated extraocular EMG activity during the VOR [e.g., (103)], because the evoked eye movements are usually too large to enable safe and stable (artifact-free) recordings from within the muscles. Sound and vibration stimuli are useful in this context as the head remains still and the evoked eye movements are extremely small (61, 104). The sharp onset of the stimuli ensures that the eye movements, and therefore the muscle contractions that underlie them, are fast and synchronous, properties that lend themselves to single motor unit recordings. Weber et al. (39) took advantage of these properties to record the excitability changes underlying oVEMPs in normal human volunteers. oVEMPs are likely to be present in any extraocular muscle that contributes to an evoked eye movement, but are not equally easy to record. The largest surface responses are recorded from beneath the eyes during up-gaze, and show clear (crossed) laterality. Clinical application of oVEMPs has therefore focused on the initial biphasic wave (n10–p15) recorded under these conditions.

Weber et al. (39) recorded from both the inferior oblique (IO) and inferior rectus (IR) muscles to determine the origin and polarity of the surface responses. Their results showed that there were vibration-evoked projections to both muscles. The initial response was excitatory and appeared at ~10.5 ms in IO and 14.5 ms in IR. In fact, in both muscles the response consisted of a series of increases and decreases of activity, offset by 4–5 ms in the IR, such that the IO was active when the IR was not, and vice versa. Thus the activity was reciprocal, consistent with the muscles being vertical antagonists. There was a similar sound-evoked increase in firing at ~13.3 ms in the IO muscle, which was limited to the contralateral eye, confirming the contralateral projection. These recordings demonstrated short-latency vestibulo-ocular projections from the otoliths to individual eye muscles for the first time in humans. They highlighted the incredible synchronicity of motor unit activity that can occur during the VOR. The duration of the effect was extremely short: in four of six motor units the duration was a single 1 ms histogram bin. The discharge at this latency was 1.8–9.9 times the baseline activity.

**Figure 7:**
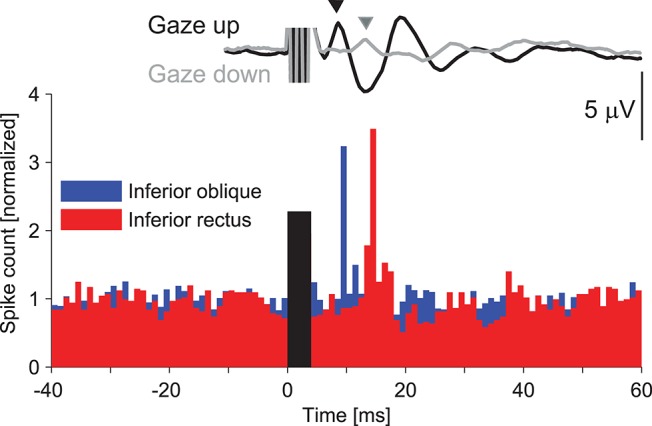
Sound evoked responses in the inferior oblique versus inferior rectus extraocular muscles. The histogram shows responses of single motor units from the IO and IR muscles of a normal volunteer in response to vibration delivered to the forehead (39). There is a series of increases and decreases in muscle activity in both muscles, beginning with an excitation, but offset in IR by about 4–5 ms. The surface oVEMP traces were recorded in a separate study (105) and represent the grand mean trace from ten normal volunteers. The oVEMP recorded during up-gaze (black trace) has an initial negative (up-going) peak at about 10 ms, which coincides with the latency of excitation in the intramuscular response from the IO. The oVEMP recorded during down-gaze (gray trace) has a smaller initial negativity that peaks at the same time as the intramuscular excitation in the IR muscle (about 14 ms). Rosengren et al. (105) showed that the vertical gaze effect on oVEMP amplitude was produced by a combination of scaling with background muscle contraction (the larger effect) and changes in proximity of the muscle to the recording electrodes (smaller effect). It is therefore likely that oVEMPs recorded during up-gaze originate in the IO and in down-gaze in the IR muscle. The histogram was reprinted with permission from Weber et al. (39). The surface responses were reprinted by permission from Rosengren et al. (105).

Similar to the cVEMP, histograms containing data from multiple (compared to single) motor units most resembled the surface response. Even though both inferior extraocular muscles were activated by the vibration stimulus, the surface response recorded from beneath the eyes appeared to be dominated by the IO response, at least during up-gaze. Rosengren et al. (105) performed a subsequent study to investigate the effect of gaze on the oVEMP and found that the surface response in up-gaze matched the multiple unit histograms from the IO muscle, while the surface response in down-gaze matched the histograms from the IR muscle (Figure 7). They showed that the vertical gaze effect on oVEMP amplitude was produced by a combination of scaling with background muscle contraction (the larger effect) and changes in proximity of the muscle to the recording electrodes (smaller effect). Thus in up-gaze, the IO is active and is closer to the surface electrodes, producing large oVEMPs that have an initial excitatory peak at ~10 ms, like the IO intramuscular response, while in down-gaze the IR is active but further away from the electrodes, producing small oVEMPs that have an initial excitatory peak at ~14 ms, like the IR intramuscular response (105).

#### Relationship of VEMPs to the linear vestibulo-collic and vestibulo-ocular reflexes

Normal head movements consist of both rotational and linear displacements. The semicircular canals provide signals of head rotation, while the otoliths respond to linear acceleration. Compensating for arbitrary movements requires a combination of both types of outputs. The cVEMP and the oVEMP occur at very short latency and are likely to consist of 3 neurone arcs. Although long term plasticity is possible, analogous to the well-known rotational vestibulo-ocular reflex, this has not been investigated to date. The end organs that appear to be excited by phasic sound and vibration stimuli are the irregularly-discharging otolith afferents (27). The cVEMP would thus represent a form of otolithic VCR and the oVEMP an otolithic VOR. The reflexes evoked by otolith afferents are termed the linear vestibulo-collic (LVCR) and linear vestibulo-ocular (LVOR) reflexes and can be initiated by either tilt or translation. The distinction from their canal-based equivalents is important. Neither otolith organ is effectively activated by head rotation, the typical stimulus used for the rotational reflexes. While the saccule is mainly excited by vertical acceleration and the utricle by accelerations in the horizontal plane, small pitches and rolls from the upright will have a greater effect on utricular rather than saccular afferents (106, 107).

##### The role of reflexes

Although stereotyped, reflexes provide the advantage of speed and, in most cases, make no demands upon cognitive resources. Phasic reflexes are ideal for correcting small perturbations in a subconscious process and priming muscles for responses to larger ones. More tonic inputs are likely to be used to determine the set point for eye and head orientation (108). Both cVEMPs and oVEMPs show the property of scaling to adapt to the level of background activation (8, 100), a simple means of ensuring that the size of the response remains equally effective despite changes in the level of ongoing activity. Physiologically, however, the utricle in particular suffers from an intrinsic ambiguity—an excitation may indicate either an ipsilateral head tilt (effect of gravity) or a contralateral head acceleration (inertial effects). It is not clear how this is resolved, although one simple method would be to assume that short-duration effects were due to imposed lateral accelerations and long duration effects due to postural changes occurring with a change in head orientation (tilt) with respect to gravity (109). Telford et al. (110) proposed that translational VOR responses were consistent with high pass filtering of LVOR pathways.

##### The cVEMP and the linear vestibulo-collic reflex

Both saccular- and utricular-based reflexes occur in the SCM, underlying the cVEMP. The short latency saccular projection as demonstrated in humans is inhibitory to the ipsilateral SCM (46, 93) and inhibitory to the contralateral splenius (41), its agonist for head rotation. This pattern suggests that unilateral saccular input may act to rotate the head toward the side excited but would not be activated by head rotation in a standing human. Bilateral saccular activation would not be expected to have any net effect for rotation but would tend to extend the neck, consistent with a role in head-righting reflexes (111). It may be that the saccular VCR is more active with the head held closer to the position for the quadruped, such as with crawling. In such a posture, differences in saccular activation may act to align the head vertically. There is also a vestibular-dependent head-dropping reflex, which occurs at a slightly longer latency than the cVEMP (112), but this may represent a labyrinthine-triggered startle reflex, mediated by reticulospinal efferents. Posturally-facilitated startle responses occur at shorter latencies than the better-known acoustic startle reflex (113).

Impulsive bone-conducted stimuli delivered in the horizontal, interaural plane are thought to be particularly selective for the utricle (114). Such impulsive responses in SCM are inhibitory ipsilaterally and excitatory contralaterally for a positive impulse to the head (positive as used here means evoking an acceleration *away* from the site of stimulation), and reversing the direction of movement inverts the response, although the initial excitation seen with surface electrodes may not be prominent (93, 115). The response to a positive perturbation is consistent with a nett excitation of the ipsilateral utricle, particularly its medial part. The findings fit with the pattern of brainstem activation initially reported by Wilson et al. (116) but not with later reports (117). The effects of impulsive stimuli have not been investigated in other neck muscles in humans. Inhibition would be the expected excitability change to compensate for an ipsilateral head tilt, perhaps imposed by lateral acceleration of the trunk during locomotion. Similar accelerations of the head do occur in normal walking (108). Other studies however suggest that the dominant utricular projection should arise from the lateral side of the striola [e.g., (118)]. Further study of the effects of utricular activation on neck muscles may provide more clarity for this projection and its effects on splenius capitis could be particularly informative.

##### The oVEMP and the linear vestibulo-ocular reflex

The rotational and linear VORs have evolved to stabilize the eyes on target, despite imposed perturbations. Corresponding to the two methods of exciting the otoliths are two possible linear otolith-ocular reflexes—one to tilt and one to translational acceleration—and this applies to perturbations both in the interaural axis and the naso-occipital axis. The oVEMP shows clear direction-dependence for its initial excitability—both for impulsive stimuli and for BC vibration, although not for similar AC stimuli (94).

Vertical accelerations evoke the translational vertical VOR (110, 119), and are presumed to arise from vestibular receptors. Saccular afferents are activated by vertical accelerations, however, short latency saccular-ocular responses appear to be weak (59), so it is not clear to what extent these linear reflexes are related to the pathways underlying oVEMPs. This may be why gains of the vertical TVOR rapidly fall in darkness (119).

Lateral tilt is a well-established method of measuring otolith function by evoking ocular counter-roll (118). In humans the gain is modest, although this is for large, low-acceleration movements. Lateral acceleration excites the translational VOR, which is most important for high frequencies and near target viewing (120). A characteristic feature of the translational VOR is that the gain changes inversely with the distance of the object (109). Aw et al. (121) found nearly exact horizontal VOR compensation in the first 100 ms in normal subjects who were laterally accelerated, with very little torsional component. They found that responses in both directions were roughly halved after unilateral lesions, implying a bilateral input to both eyes. Todd et al. (65) showed differential effects of lateral acceleration on oVEMPs recorded from laterally-placed surface electrodes, consistent with excitation of the lateral rectus muscle on the side of the applied positive acceleration. Interestingly, Govender et al. (64) reported bilateral attenuation of oVEMPs in lateral electrodes in patients with unilateral lesions, in contrast to the crossed unilateral attenuation seen with conventional inferior electrodes recording from the inferior oblique muscles. This finding supports a role for both the medial and lateral parts of each utricle in this form of the reflex. The lack of torsion recorded by Aw et al. (121) might be a consequence of the eyes being in the neutral position, as the responses from the inferior oblique muscles increase with up-gaze. Telford et al. (110) also used interaural translations and found that small torsional responses were generated at the same time as horizontal responses and these had little relationship to vergence angle. A better relationship was found with tilt gain. It is possible therefore that the conventional oVEMP recorded from beneath the eyes relates to ocular torsion evoked by a tilt-VOR, while a translational-VOR effect can be detected when using electrodes lateral to the eyes.

Antero-posterior translations should also evoke linear VORs. For anterior translational acceleration, one would expect convergence bilaterally, with the opposite for posterior accelerations. Forward acceleration should induce the same change in the otolith discharge as head elevation. For head elevation the inferior oblique muscles should be inhibited if to be compensatory. With the eyes neutral the main effect of the translational VOR should be convergence-divergence, but with the eyes elevated implies relative intorsion (122). Todd et al. (123) showed oVEMPs evoked by antero-posterior movements in subjects with their eyes elevated once the movements were over 4 Hz and argued that the oVEMP was a manifestation of the LVOR. Govender and Colebatch (124, 125) found oVEMP excitability changes consistent with anterior acceleration causing excitation, and posterior acceleration causing inhibition, as expected for the translational VOR.

### Otolith sensitivity

A novel application of VEMPs has been to test the sensitivity of the otolith organs to linear acceleration. The traditional method of measuring otolith sensitivity is by measuring thresholds to linear translations of the head and body, which can be cumbersome and potentially affected by other sensory systems such as proprioception. In contrast, BC vibration applied to the skull is benign method for activating the otolith organs, and lends itself to detection of small reflexes over many repetitions. Todd et al. (126) explored frequency tuning of the BC oVEMP and found that a 100 Hz, 50 ms stimulus applied to the mastoid (directed interaurally) produced a large reflex that could be used to test the sensitivity of the otoliths to vibration. By systematically reducing the stimulus intensity and averaging over several thousand repetitions from four volunteers, Todd et al. (126) recorded oVEMPs with skull acceleration as low as 70.2 dB below 1 g (around 0.0003 g, 0.303 cm/s^2^). This stimulus intensity was below the level that subjects could hear or feel. It is below the average perceptual thresholds reported in normal human volunteers in response to linear translation in the sway (interaural) direction (about 2–7 cm/s^2^), but possibly close to the lower limits of normal (127–129). Compared to the thresholds recorded to BC vibration in some species of frog, e.g., 90–120 dB below 1 g, the reflex thresholds in humans are much higher (126, 130). However, thresholds estimated from reflexes may underestimate the sensitivity of an organ. For example, the difference between auditory brainstem reflex thresholds and behavioral thresholds tested with pure tone audiometry are around 15–20 dB at 500 Hz (131). Taking this into account, the data from Todd et al. (126) suggest that the sensitivity of human otoliths approaches that of the otolith organs of frogs.

## VEMP-inspired studies in patients

### Central vestibulopathy

If VEMPs are otolith reflexes, then an abnormal or absent VEMP indicates a problem with otolith receptors or the pathway of the reflex. Unless there is recovery of function (as can happen in vestibular neuritis, for example) VEMPs remain abnormal even after central vestibular compensation has occurred. In this respect VEMPs are different to subjective visual horizontal or vertical, another test of otolith function, which returns to normal as the resting firing rate in the brainstem vestibular nuclei normalizes.

VEMP studies in patients with brainstem lesions have provided supporting evidence in humans for the locations of central otolith projections. The otoliths project directly to the vestibular nuclei in the medulla and pons, mainly to the lateral and descending vestibular nuclei (34, 132). Saccular projections are predominantly to the descending nucleus and the ventral part of the lateral vestibular nucleus, with some projections to the medial vestibular nucleus (132–135). In contrast, utricular fibers project more evenly within the vestibular nuclei: throughout the descending nucleus, in the lateral part of the medial nucleus, in the lateral nucleus and ventral part of the superior nucleus (34, 134, 136, 137). The vestibulospinal tracts consist of both medial (MVST) and lateral (LVST) components, which originate in the vestibular nuclei in the medulla and pons; fibers in the MVST arise from the medial, descending and lateral nuclei and fibers in the LVST arise in the lateral vestibular nucleus (34, 138). The MVST descends in the medial longitudinal fasciculus (MLF) bilaterally, while the LVST descends ipsilaterally in the ventral funiculus. Both the MVST and LVST project directly and indirectly to neck motoneurons, while progressively fewer neurons extend to the lower cervical, thoracic and lumbar spine levels, and these neurons are thought to course through the LVST (138–140). In contrast, vestibulo-ocular projections originate mainly in the medial and superior vestibular nuclei and ascend within the MLF to the oculomotor nuclei (57).

Patient studies in humans have largely been consistent with these projections. cVEMPs tend to be abnormal when the lesion affects the vestibular nuclei (in particular the lateral nucleus), spinal accessory nucleus and areas of the MLF in between (141–145). For example, both medial and lateral medullary infarction have been associated with significant rates of cVEMP abnormality (142, 143, 146). In contrast, cVEMPs tend to be spared in patients with lesions above the vestibular nucleus (146), but oVEMPs are often abnormal in these patients (147). For example, patients with MLF lesions producing internuclear ophthalmoplegia have high rates of oVEMP abnormality and low rates of cVEMP abnormality (148, 149). Overall, there are only moderate rates of VEMP abnormality in patients with brainstem lesions (up to about 50%) and there is only modest concordance between imaging and VEMP abnormalities. This may be partly due to timing effects, whereby ischaemic lesions may appear smaller in the acute phase, and the fact that functional impairments may be more extensive than obvious structural lesions defined on imaging (143). There are also indirect vestibular pathways within the brainstem and neurons which project to both the vestibulo-ocular and vestibulospinal systems (138), which may help account for some of the VEMP abnormalities seen with lesions outside the known primary pathways.

Similar to other evoked potentials, in patients with known central pathology VEMPs can help localize central lesions. An isolated absent cVEMP would place the lesion at or below the vestibular nucleus in the MVST or spinal accessory nucleus, an absent oVEMP would indicate a lesion at or above the vestibular nucleus in the MLF or oculomotor nucleus, and a combined cVEMP/oVEMP abnormality would suggest a lesion in the vestibular nucleus or root entry zone.

### Superior canal dehiscence

Aside from detecting loss of function of the otoliths or their pathways through the brainstem, VEMPs are sensitive to changes in the mechanical properties of the vestibule. This is because sound and vibration stimuli interact in specific ways with the bony and membranous structures in the labyrinth on their way to activating the receptors. In the case of AC sound, the peripheral auditory structures are organized to move sound efficiently to the cochlea without affecting the nearby vestibular organs. Only very loud sounds can breach this system and act as a vestibular stimulus, and this is why VEMP thresholds are normally so much higher than hearing thresholds. In the case of BC vibration, variables affecting the pathway of the stimulus include the site and direction of the stimulus, as well as individual variations in skull shape and orientation of otolith membranes. Despite this variability, a change in the structure of the labyrinth, as in SCD, can also produce systematic changes in the movement of vibration through the vestibule.

In SCD a dehiscence produces a third mobile window in the labyrinth, allowing sound energy that would not normally enter the labyrinth to do so. Signs of increased vestibular sensitivity to sound (i.e., Tullio phenomenon) include sound-evoked nystagmus and enhanced postural reflexes (head movements/jerks), both in the plane of the affected superior semicircular canal. VEMPs are a direct reflection of these enhanced reflexes: cVEMPs in SCM contribute to the muscle activity that produce the head jerk and oVEMPs in IO contribute to the extraocular muscle contractions that cause the sound-evoked eye movements. Along with observation of these signs, VEMPs provide a means of confirming that a hole seen on imaging has a physiological effect (150). Computed tomography scans can provide false positive results, where there is thin bone covering the superior canal, and for this reason it is recommended that a physiological test such as the VEMP be included in diagnosis (150). VEMPs may also provide an indication of the size of the hole: the length and area of dehiscence as measured radiologically correlate with cVEMP threshold (151–153) and sometimes also cVEMP and oVEMP amplitude (151). However, some studies have not shown this relationship, possibly because the dehiscence was measured during surgery, meaning that patients with milder symptoms, who may have had smaller holes, were potentially omitted (154, 155). VEMPs evoked by BC vibration also seem to be systematically altered in SCD. Stimulation at the forehead produces responses with delayed latency (156) and stimulation at the vertex produces abnormally large reflexes compared to normal controls (156, 157). Finally, VEMPs can also demonstrate that a dehiscence has been effectively fixed. Welgampola et al. (158) showed that thresholds and amplitudes of both cVEMPs and oVEMPs to AC and BC stimuli return to normal after plugging the canal [see also (155)].

### Meniere's disease

VEMPs are also sensitive to the alterations in fluid and pressure in the vestibule in Meniere's disease (MD). In normal ears, the sound-evoked cVEMP and oVEMP tend to have a preferred tuning of around 500 Hz (159–163), although the oVEMP naturally prefers a slightly higher frequency than the cVEMP (164). However, in ears affected by Meniere's disease, the preferred frequency has been shown to shift upwards toward 1,000 Hz for both reflexes (164–170). It has now been shown that comparing the responses at these frequencies in the form of a ratio is helpful in distinguishing MD from other causes of vertigo (169, 171). These changes in tuning were demonstrated in patients tested between attacks, suggesting that the changes are reasonably stable over time. It has also been shown that VEMPs are modulated *during* an attack of MD. Manzari et al. (172) showed that oVEMPs evoked by skull vibration increase in size during an attack, while cVEMPs decrease. Another characteristic finding in early Meniere's disease is the enhancement of the VEMP despite the presence of a canal paresis (173). This paradoxical increase presumably indicates an increase in sound transmission to the (congested) saccule. It thus appears that the disease systematically changes the resonance of the system, presumably due to endolymphatic hydrops in the labyrinth changing pressure and stiffness, though the exact cause of this is not known (168, 174).

### Neurodegenerative disease

Otolith afferents have extensive cortical projections and these have been demonstrated using techniques to activate otolith receptors (88, 92). A recent unexpected finding has been impairment of VEMPs in patients with neurodegenerative diseases such as dementia (175, 176). For example, Harun et al. (175) showed that patients with Alzheimer's disease had significantly smaller and more frequently absent cVEMPs and oVEMPs than a large sample of age-, gender-, and education-matched controls, while VOR gain on the video head impulse test was not affected. VEMP abnormalities have also been found in patients with Parkinson's disease, and are correlated with both motor and non-motor effects of the disease (177–181). It is possible that the concomitant decline in otolith function in patients with neurodegenerative disease is a sign of more widespread decline in neural function impacting the brainstem. However, medication and age effects have not been excluded in some of the above studies.

## Conclusion

After 25 years of the VEMP, we now have a much clearer understanding of the effects of sound and vibration on the vestibule. Studies in both animals and humans have shown that these stimuli are quite selective for irregularly-firing otolith afferents, and that AC sound activates the saccule more than any other vestibular organ. The properties of sound and vibration, including their brevity and suitability for repetitive stimulation, have enabled them to be applied in normal human volunteers to investigate the effects of otolith activation. Using these stimuli, studies have shown that the otoliths project to postural muscles throughout the body, from the eyes to the lower legs. Otolithic projections to the brain have also been demonstrated. Detailed studies of the cVEMP and oVEMP have provided information about the brief excitability changes that occur in the neck and eye muscles following sound and vibration stimulation and show how they relate to the responses recorded at the surface. Along with its clinical utility, in particular in SCD, the VEMP has therefore provided interesting opportunities, both directly and indirectly, to investigate vestibular otolith reflexes in normal human subjects.

## Author contributions

SR drafted and edited the manuscript. JC drafted part of the manuscript and edited it.

### Conflict of interest statement

The authors declare that the research was conducted in the absence of any commercial or financial relationships that could be construed as a potential conflict of interest.
